# ALT Flare Predicts Hepatocellular Carcinoma Among Antiviral Treated Patients With Chronic Hepatitis B: A Cross-Country Cohort Study

**DOI:** 10.3389/fonc.2020.615203

**Published:** 2021-01-21

**Authors:** Yanan Du, Bingying Du, Xiaoyan Fang, Meng Shu, Yongjing Zhang, Hsingwen Chung, Ye Sun, Jiaming Teng, Phimphone Visalath, Hong Qiu, Wei Cai

**Affiliations:** ^1^ Department of Infectious Diseases, Ruijin Hospital, Shanghai Jiao Tong University School of Medicine, Shanghai, China; ^2^ Department of Epidemiology, Janssen Research and Development, Shanghai, China; ^3^ Global Epidemiology, Janssen Research and Development, Titusville, NJ, United States

**Keywords:** alanine aminotransferase flare, hepatocellular carcinoma, risk factor, chronic hepatitis B, nucleos(t)ide analog

## Abstract

**Objectives:**

Alanine aminotransferase (ALT) level is one of the crucial indexes to evaluate disease status for chronic hepatitis B (CHB) patients. However, whether the ALT level after nucleos(t)ide analog (NA) treatment is associated with hepatocellular carcinoma (HCC) development remains unclear.

**Materials and Methods:**

We evaluated the association between ALT level and HCC occurrence in NA-treated patients and investigated the predictive value of ALT flare for HCC. The associations between ALT level and HCC were analyzed by logistic regression and Cox proportional hazards models.

**Results:**

There were 21,223 CHB patients at Ruijin Hospital of China and 16,737 CHB patients in the Optum electronic health records (EHR) in the United States (US) treated with NAs between 2010 and 2018. Among them, 8,152 and 4,893 patients who achieved a normal ALT value were included in the study cohorts, respectively. A significant positive dose-dependent correlation between the peak ALT level and HCC was identified in both cohorts. Within the China cohort, ALT flare was significantly associated with increased risks of HCC compared to normal ALT (HR 2.55, 95%CI 1.45–4.50). Stronger increased risks associated with ALT flare were observed in the US cohort (HR 7.62, 95%CI 4.85–11.98).

**Conclusions:**

ALT flare is a strong predictor for HCC occurrence in the CHB patients treated with NAs. Elevation of ALT, especially ALT flare warrants close monitoring for early HCC detection.

## Introduction

An estimated 240 million people are chronically infected with hepatitis B virus (HBV) worldwide, with the highest prevalence of infection in Asia (1). Chronic hepatitis B (CHB) progresses to cirrhosis, liver failure, or hepatocellular carcinoma (HCC) in 15–40% of infected people (2). Treatment with oral nucleos(t)ide analogs (NAs) can induce long-term inhibition of HBV replication in patients with CHB, leading to regression of cirrhosis, prevention of hepatic decompensation, and a reduction in the risk of HCC (3). However, among well controlled NA treated CHB patients, some of them still develop HCC. In China, HCC is the fourth most common cancer and the third leading cause of cancer-related death (4).

Significant increases in alanine aminotransferase (ALT) can occur during the natural history of CHB and during treatment with NAs. While ALT elevation may be associated with seroclearance of HBV DNA and HBV antigens under NA treatment as well as during the progression of the disease (5, 6), other evidence suggests that elevated ALT in CHB patients without antiviral treatment at baseline can be associated with a significantly increased risk for developing HCC. In a study from a Hong Kong cohort of 723 CHB patients with HBeAg seroclearance, patients with persistently abnormal ALT had a >6-fold increased risk of HCC compared to patients with persistently normal ALT (7). Evidence from a large HBV cohort study, the Risk Evaluation of Viral Load Elevation and Associated Liver Disease/Cancer in HBV (REVEAL-HBV) in Taiwan yielded similar results that elevated ALT was associated with a >five-fold increased risk of HCC among patients with CHB and HBV carriers (8, 9). However, another study of 588 Korean American CHB patients did not observe a significant association between ALT and HCC risk (10). As yet, it is not clear whether or how, ALT elevations are associated with HCC, especially among those with NA treatment.

The standard of normal for ALT remains contentious (11). There is marked variability in the upper limit of normal (ULN) for ALT in clinical practice guidelines published by the European Association for Study of the Liver (the traditional 40 U/L) (12, 13), and the American Association for the Study of Liver Diseases (AASLD) (35 U/L for males and 25U/L for females) (14). In an Asian study, ULNs of ALT in healthy individuals were 33 U/L for men and 25 U/L for women (15). In a South Korean study, the ALT cut-off for HCC risk in the general population was proposed as 16.5 U/L (16). Furthermore, there is no convincing evidence to prove that the ULN applied to the general population is the most suitable for the evaluation of patients with CHB (11).

We conducted a large retrospective cohort database study in China to investigate potential associations between severe ALT elevation, so called “ALT flare” and the risk of HCC among CHB patients whose ALT levels had normalized after treatment with NA. In addition, we conducted the same analyses using an electronic health record (EHR) claims database in the United States (US) to support our conclusion.

## Materials and Methods

### Data Sources

This study was conducted using two electronic healthcare databases. One is an electronic medical records (EMR) database from Ruijin Hospital, a tertiary general hospital in Shanghai, China. The other is the Optum PanTher EHR database in the US. Details about these data sources are described in the supplementary document. The study conducted in the Ruijin database was approved by the Human Ethics Committee, Ruijin Hospital, Shanghai Jiao Tong University School of Medicine, and the analysis on the Optum database was exempt from an institutional review board (IRB) approval by the New England IRB. Patient consent was not required for the use of de-identified, electronic data consistent with Health Insurance Portability and Accountability Act (HIPAA) (17).

### Study Population

**Figure 1:**
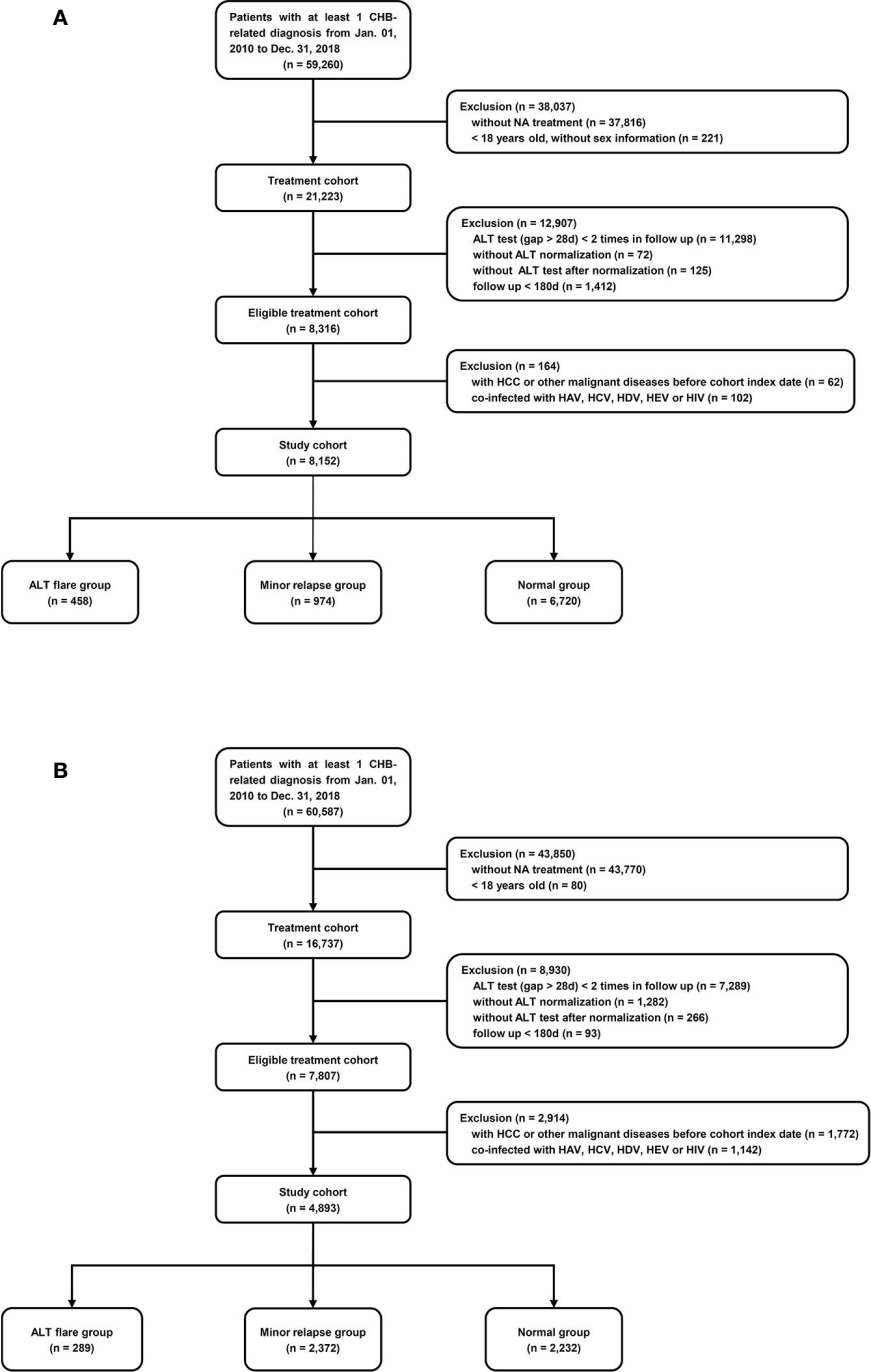
Cohort selection. **(A)** Ruijin Hospital, China. ALT flare group: ALT relapsed to ≥128 U/L; minor relapse group: ALT relapsed to 64**–**128 U/L; normal group: ALT achieved and maintained ≤64 U/L. **(B)** Optum EHR database, the US. ALT flare group: ALT relapsed to ≥175 U/L in men and ≥125 U/L in women; minor relapse group: ALT relapsed to 35**–**175 U/L in men, 25**–**125 U/L in women; normal group: ALT achieved and maintained ≤35 U/L in men, ≤25 U/L in women.

The study population included all patients with at least one diagnosis of CHB and who had been prescribed NA during January 01, 2010 to December 31, 2018. The Chinese cohort was extracted from the hospital medical records system at the Ruijin Hospital using relevant ICD-10 codes as well as text diagnoses. The US cohort was identified as those patients with a diagnostic code of CHB (ICD9 = 070.32 and ICD10 = B18.1) in the Optum EHR US database. Individual records of all HCC diagnoses in both cohorts were manually reviewed by a physician based on patients’ imaging manifestation (computed tomography or magnetic resonance) and biopsy results. Included subjects were at least 18 years of age at the first identified NA treatment; had at least twice ALT tests with at least 28 days apart after NA treatment; was once normalized after treatment initiated and followed longer than 180 days. Patients with a diagnosis of HCC or any other malignancy, or infection with hepatitis A, C, D, E viruses or human immunodeficiency virus (HIV) at, or prior to, the cohort index date was excluded (**Figure 1**).

### Exposure Groups: Definition of ALT Pattern

All ALT values were identified from the laboratory results during the cohort follow-up. The peak ALT values were evaluated to identify ALT flare and the average of the three highest ALT values were categorized as ≤25, 25–40, 41–64, 65–80, 81–128, and >128 U/L.

The ULN of the local hospital standard for ALT was 64 U/L in China. An ALT flare was defined as an increase in ALT to ≥128 U/L (2× local ULN) and a ‘minor relapse’ was defined as an ALT level >64 but <128 U/L. The ULN defined by the AASLD: 35 U/L for men and 25 U/L for women (14) was substantially lower than the local ULN in China. An ALT flare in the US cohort was defined as 5× AASLD ULN (≥175 U/L in men and ≥125 U/L in women), which is approximately the same level as a two-fold increase in the local ULN in China. The minor relapse was defined as an ALT level rise to >35 but <175 U/L in men, >25 but <125 U/L in women. The ‘ALT elevation’ group referred to all patients who experienced either an ALT flare or a minor relapse, while ‘normal’ referred to patients whose ALT stayed at or under the ULN of their relevant standards, after their first normalization.

### Study Cohorts

The cohort entry date was the earliest date of a patient achieving normal ALT criteria (1^st^ ALT normalization date) with NA treatment. The flare index dates were defined as the earliest date the ALT increased to meet the flare definition or the minor relapse definition, respectively. For patients who maintained normal levels of ALT, the cohort index date was defined as the date of highest ALT level after first normalization. The baseline period was defined as the 12-month period prior to, and including, the cohort index date. Person–years of follow-up were calculated from the cohort index date until the date of first diagnosis of HCC, or the end of follow-up if no HCC occurred. Patients were followed until the last healthcare visit date plus 45 days, or December 31, 2018, whichever occurred first.

### Statistical Analysis

In the two study cohorts, incidence rates of HCC occurrence were estimated. The association between peak ALT value, as the average of the three highest ALT values after the cohort entry and before the follow up (HCC or end of study, whichever is earlier), was analyzed using a logistic regression model. Odds ratios, 95% CIs, and *p*-value for trends were estimated. Univariate analyses were conducted for all the potential confounders, which were age, gender, prior cirrhosis, prior liver failure, baseline diagnoses of hypertension/diabetes, alcoholic liver disease, nonalcoholic fatty liver disease (NAFLD), NA treatment duration prior to the flare index date and whether patients were on treatment at the flare index day. The potential confounders were retained in the final model only if their inclusion changed the OR for the ALT flare by 10% or more, relative to the unadjusted OR for the ALT flare.

For the association between ALT flare and HCC, a Cox proportional hazards model was applied to investigate potential associations between ALT flare and the development of HCC by estimating hazard ratios (HR) and their 95% CIs. Univariate Cox regression were conducted for all the potential confounders, same as mentioned in the logistic model construction. The potential confounders were retained in the final model only if their inclusion changed the HR for the ALT flare by 10% or more, relative to the unadjusted HR for the ALT flare (*i.e.* adjusted HR/unadjusted HR is either >1.10 or <0.90), the so-called ‘change in estimate’ criterion (18). Kaplan–Meier curves were also constructed.

As drug resistance was shown as an impact factor of ALT flare and subsequent virological breakthrough, which may influence the development of HCC (19, 20), it might confound the association between ALT flare and HCC. However, only a subset of the patient in China cohort had drug resistance tests, and no test data is available in the US cohort due to the nature of the claim data sources. Thus, we conducted a sensitivity analysis to repeat the main analyses among a subset of patients received drug resistance tests in the China cohort.

All analyses on Ruijin Hospital data were conducted using R 3.6. For the analyses in the US Optum EMR database, most were conducted using SAS enterprise guide v 7.15 in addition to using R 3.6.

## Results

### Demographic and Clinical Characteristics of the Study Cohort in Ruijin Hospital, China

A study cohort of 8,152 patients was identified among 21,223 adult patients with CHB who were treated with NA at Ruijin Hospital, China (**Figure 1A**). The mean (± SD) age was 44.7 (± 13.8) years, and 5,559 (68%) were male, 1,463 (18%) had cirrhosis, 357 (4.4%) had NAFLD, 462 (5.7%) had alcoholic liver disease (**Table 1**). There were 458 patients (6%) who experienced ALT flares, 974 (12%) who experienced minor ALT relapses, and 6,720 (82%) who maintained at the normal ALT over the follow-up (**Figure 1A**). During the mean follow-up of 1.81 years, a total of 103 patients were diagnosed HCC (incidence, 7.0/1000 person-years). The main baseline characteristics of respective groups were presented in **Table 1**. The mean durations of follow-up were 718, 724, and 646 days in ALT flare, minor relapse and normal groups, respectively. The mean durations of NA treatment prior to the ALT index date were 389, 311 and 329 days in ALT flare, minor relapse, normal groups, respectively (**Table 1**).

**Table 1:** Baseline demographic and clinical characteristics of study cohort in the Ruijin Hospital, China.

Characteristics	Study cohort	ALT flare	Minor relapse	Normal
N (%)	8,152	458 (5.6)	974 (12.0)	6,720 (82.4)
Age, years (Mean ± SD)	44.7 ± 13.8	44.5 ± 13.7	42.3 ± 13.0	45.1 ± 13.8
18–30 (%)	1,200 (14.7)	64 (14.0)	177 (18.2)	959 (14.3)
31–40 (%)	2,371 (29.1)	135 (29.5)	321 (33.0)	1,915 (28.5)
41–50 (%)	1,718 (21.1)	96 (21.0)	210 (21.6)	1,412 (21.0)
51–60 (%)	1,506 (18.5)	90 (19.7)	140 (14.4)	1,276 (19.0)
>60 (%)	1,357 (16.6)	73 (15.9)	126 (12.9)	1,158 (17.2)
Gender, Male (%)	5,559 (68.2)	350 (76.4)	788 (80.9)	4,421 (65.8)
Baseline HBV DNA, IU/ml				
<2,000 (%)*	6,655 (84.9)	301 (68.3)	702 (74.5)	5,652 (87.6)
≥2,000 (%)*	1,181 (15.1)	140 (31.7)	240 (25.5)	801 (12.4)
Baseline HBeAg				
Negative (%)*	3,947 (53.9)	212 (49.0)	425 (46.7)	3,310 (55.3)
Positive (%)*	3,381 (46.1)	221 (51.0)	486 (53.3)	2,674 (44.7)
Cirrhosis				
No (%)	6,689 (82.1)	379 (82.8)	772 (79.3)	5,538 (82.4)
Yes (%)	1,463 (17.9)	79 (17.2)	202 (20.7)	1,182 (17.6)
Liver failure				
No (%)	7,684 (94.3)	417 (91.0)	882 (90.6)	6,385 (95.0)
Yes (%)	468 (5.7)	41 (9.0)	92 (9.4)	335 (5.0)
NAFLD				
No (%)	7,795 (95.6)	428 (93.4)	902 (92.6)	6,465 (96.2)
Yes (%)	357 (4.4)	30 (6.6)	72 (7.4)	255 (3.8)
Alcoholic liver disease				
No (%)	7,690 (94.3)	432 (94.3)	922 (94.7)	6,336 (94.3)
Yes (%)	462 (5.7)	26 (5.7)	52 (5.3)	384 (5.7)
Baseline biopsy				
No (%)	8,089 (99.2)	454 (99.1)	965 (99.1)	6,670 (99.3)
Yes (%)	63 (0.8)	4 (0.9)	9 (0.9)	50 (0.7)
Hypertension				
No (%)	7,995 (98.1)	445 (97.2)	946 (97.1)	6,604 (98.3)
Yes (%)	157 (1.9)	13 (2.8)	28 (2.9)	116 (1.7)
Diabetes				
No (%)	8,029 (98.5)	446 (97.4)	952 (97.7)	6,631 (98.7)
Yes (%)	123 (1.5)	12 (2.6)	22 (2.3)	89 (1.3)
Baseline NA type (%)				
Adefovir	508 (6.2)	36 (7.9)	74 (7.6)	398 (5.9)
Entecavir	4,594 (56.4)	208 (45.4)	488 (50.1)	3,898 (58.0)
Lamivudine	1,125 (13.8)	82 (17.9)	158 (16.2)	885 (13.2)
Telbivudine	886 (10.9)	52 (11.4)	103 (10.6)	731 (10.9)
Tenofovir	365 (4.5)	27 (5.9)	54 (5.5)	284 (4.2)
Combined NAs	674 (8.3)	53 (11.6)	97 (10.0)	524 (7.8)
NA duration, days (Mean ± SD)	327.2 ± 406.2	388.6 ± 476.2	311.2 ± 404.8	328.7 ± 410.5
Treatment situation at index date (%)				
Off treatment	1287 (15.8)	97 (21.2)	159 (16.3)	1,031 (15.3)
On treatment	6,865 (84.2)	361 (78.8)	815 (83.7)	5,689 (84.7)

N, number of patients; SD, standard deviation; HBV, hepatitis B virus; HBeAg, hepatitis e antigen; ALT, alanine aminotransferase; NAFLD, nonalcoholic fatty liver disease; NA, nucleos(t)ide analog.

*Percentages were based on non-missing totals of patients with HBV DNA and HBeAg test results.

### Correlation Between the Peak ALT Level and HCC in the China Cohort

There were 6,313 patients in the China cohort with at least three ALT values after normalization following NA treatment, and 74 patients were diagnosed HCC during the follow-up. There was a positive, dose-dependent correlation between the peak serum ALT level and HCC occurrence, reaching statistical significance in trend test with *p*-values <0.001. After adjustment for age, gender, baseline cirrhosis, hypertension/diabetes, baseline NA duration and treatment situation at index date, the significant trend remained (**Table 2**).

**Table 2:** Logistic regression model of the association between the peak ALT level* and hepatocellular carcinoma in China and in the US.

Peak ALT, IU/L	N	HCC	Univariable OR (95% CI)	Multivariable** OR (95% CI)
**Ruijin Hospital, China**				
N	6,313	74		
≤25	2,026	12	–	–
25–40	2,097	23	1.86 (0.94–3.88)	1.42 (0.71–3.00)
41–64	1,298	18	2.36 (1.14–5.05)	1.95 (0.92–4.27)
65–80	297	6	3.46 (1.20–8.98)	2.93 (0.99–7.85)
81–128	324	6	3.17 (1.10–8.22)	2.85 (0.97–7.58)
>128	271	9	5.77 (2.33–13.75)	4.41 (1.73–10.89)
***p*_Trend_**			<0.001	<0.001
				
**Optum EHR database, the US**				
N	4,560	148		
≤25	1,523	23	–	–
25–40	1,803	45	1.67 (1.01–2.77)	1.39 (0.83–2.33)
41–64	698	26	2.52 (1.43–4.46)	2.11 (1.18–3.78)
65–80	136	8	4.08 (1.79–9.30)	2.93 (1.26–6.84)
81–128	152	13	6.10 (3.02–12.31)	4.72 (2.28–9.78)
>128	248	33	10.01 (5.77–17.38)	6.84 (3.85–12.15)
***p*_Trend_**			<0.001	<0.001

ALT, alanine aminotransferase; HCC, hepatocellular carcinoma; OR, odds ratio; CI, confidence interval; N, number of patients in the group.

*The peak ALT level was the average of the top 3 ALT level during the cohort follow up for each patient. Patients who had at least 3 ALT test results during the follow up were included in the analyses.

**Age, gender, baseline cirrhosis and hypertension/diabetes, baseline nucleos(t)ide analogs duration and treatment situation at index date were included in the multivariable model in China. Age, gender, baseline cirrhosis, alcoholic liver disease and hypertension/diabetes or not were included in the multivariable model in the US.

### ALT Elevation, Especially ALT Flare Was a Strong Predictor for HCC in the China Cohort

**Figure 2:**
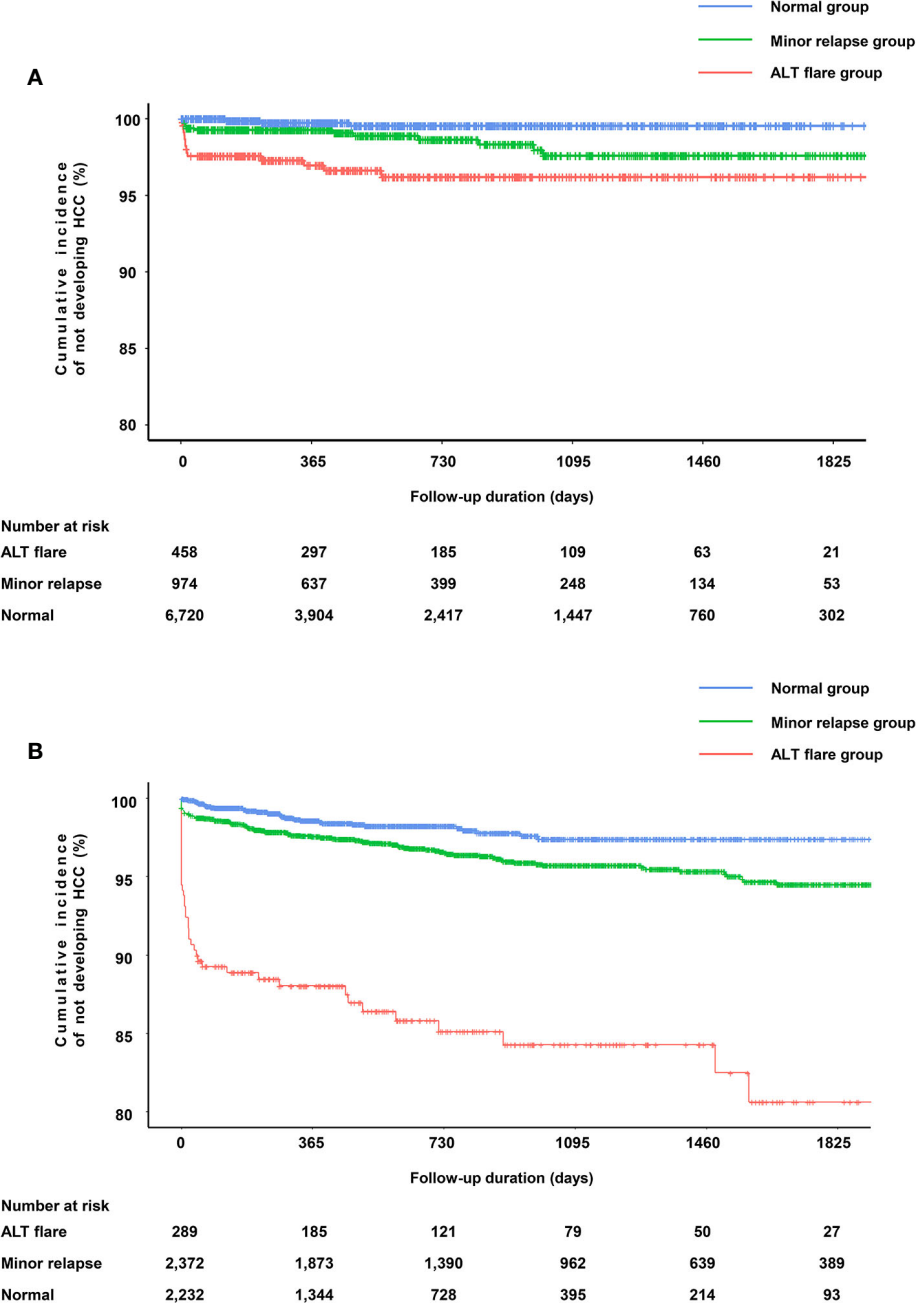
Kapan–Meier curves for HCC according to the ALT pattern. **(A)** Probability of not developing HCC in the cohort from Ruijin Hospital, China. **(B)** Probability of not developing HCC in the cohort from Optum EHR database, the US.

Among the entire study cohort of China (8,152), there were 103 cases of HCC, 15 in the ALT flare group, 17 in the minor relapse group, and 71 in the normal group (**Table 3**). ALT elevation (combined ALT flare and minor relapse groups) showed an elevated risk of HCC (adjusted HR 1.82, 95%CI 1.19–2.79) compared to the normal group (**Supplementary Table 1**). Specifically, the difference of HCC risk between minor relapse and normal groups was not statistically significant. ALT flare was associated with a significantly increased risk of HCC compared to the normal group, which remained statistically significant after adjusting for age, gender, baseline cirrhosis and diabetes/hypertension (crude HR 2.96, 95%CI 1.70–5.17 and adjusted HR 2.55, 95%CI 1.45–4.50, respectively) (**Table 3**). As observed for the overall cohort, ALT flare was associated with increased risk of HCC compared to the normal group in both men and women (**Supplementary Table 2**). The Kaplan–Meier curve confirmed a higher probability of HCC in ALT flare group compared to minor relapse and normal groups in 5 years (**Figure 2A**).

**Table 3:** Cox proportional hazard model of the association between ALT flare and hepatocellular carcinoma in China cohort.

	N	HCC	Univariable HR (95% CI)	*p*	Multivariable HR (95% CI)	*p*
N	8,152	103				
ALT pattern						
Normal	6,720	71	–	–	–	–
Minor relapse	974	17	1.56 (0.92–2.65)	0.100	1.46 (0.86–2.50)	0.164
ALT flare	458	15	2.96 (1.70–5.17)	<0.001	2.55 (1.45–4.50)	0.001
Age						
<40	3,351	10	–	–	–	–
40-60	3,264	43	4.08 (2.05–8.12)	<0.001	3.30 (1.64–6.65)	0.001
>60	1,537	50	10.05 (5.09–19.82)	<0.001	8.57 (4.23–17.36)	<0.001
Gender						
Male	5,559	86	–	–	–	–
Female	2,593	17	0.39 (0.23–0.66)	<0.001	0.34 (0.20–0.57)	<0.001
Cirrhosis						
No	6,689	55	–	–	–	–
Yes	1,463	48	3.97 (2.69–5.84)	<0.001	2.45 (1.64–3.66)	<0.001
Diabetes/hypertension						
No	7,892	89	–	–	–	–
Yes	260	14	4.73 (2.69–8.30)	<0.001	2.00 (1.11–3.60)	0.020
NAFLD						
No	7,795	102	–	–		
Yes	357	1	0.22 (0.03–1.57)	0.131		
NAs duration, days						
≤180	4,095	57	–	–		
181-365	1,519	22	1.03 (0.63–1.68)	0.921		
>365	2,538	24	0.70 (0.43–1.12)	0.136		
Alcoholic liver disease						
No	7,690	94	–	–		
Yes	462	9	1.50 (0.75–2.97)	0.248		
Liver failure						
No	7,684	94	–	–		
Yes	468	9	1.49 (0.75–2.95)	0.255		
Treatment situation at index date						
Off treatment	1,287	11	–	–		
On treatment	6,865	92	1.18 (0.63–2.21)	0.615		
Baseline biopsy						
No	8,089	103	–	–		
Yes	63	0	0.00 (0.00–Inf)	0.994		

ALT, alanine aminotransferase; HCC, hepatocellular carcinoma; HR, hazard ratio; CI, confidence interval; N, number of patients in the group; NAFLD, nonalcoholic fatty liver disease; NA, nucleos(t)ide analog.

*ALT pattern, age, gender, baseline cirrhosis, liver failure, NAFLD, alcoholic liver disease, hypertension/diabetes, biopsy, NA treatment duration and treatment situation on index date were analyzed in the model. Only those factors whose p value <0.1 remained in the final multivariable model.

### Demographic and Clinical Characteristics of the Study Cohort in the Optum EHR Database, the US

The US study cohort (4,893) was derived according to our inclusion and exclusion criteria from a total of 16,737 CHB patients (**Figure 1B**). The mean ( ± SD) age was 50.4 ( ± 13.3) years, and 3,018 (62%) were male, 807 (17%) had cirrhosis, 567 (11%) had NAFLD, 178 (3.6%) had alcoholic liver disease (**Table 4**). During the mean follow-up of 2.3 years, a total of 172 patients were diagnosed with HCC (incidence rate, 15.3/1,000 person–years). The main baseline characteristics of the respective groups are presented in **Table 4**. The mean duration of follow-up was 898, 1,054, and 639 days in ALT flare, minor relapse and normal groups, respectively. The mean durations of NA treatment prior to the ALT index date were 725, 442, and 621 days in ALT flare, minor relapse, normal groups, respectively (**Table 4**).

**Table 4:** Baseline demographic and clinical characteristics of study cohort in the Optum EHR database, the US.

Characteristics	Study cohort	ALT flare	Minor relapse	Normal
N (%)	4,893	289 (5.9)	2,372 (48.5)	2,232 (45.6)
Age, years (Mean ± SD)	50.4 ± 13.3	51.1 ± 12.8	49.6 ± 13.0	51.1 ± 13.6
18–30 (%)	336 (6.9)	23 (8.0)	175 (7.4)	138 (6.2)
31–40 (%)	889 (18.2)	47 (16.2)	441 (18.6)	401 (18.0)
41–50 (%)	1,211 (24.8)	57 (19.7)	622 (26.2)	532 (23.8)
51–60 (%)	1,324 (27.1)	84 (29.1)	648 (27.3)	592 (26.5)
>60 (%)	1,133 (23.1)	78 (27.0)	486 (20.5)	569 (25.5)
Gender, Male (%)	3,018 (61.7)	189 (65.4)	1,407 (59.3)	1,422 (63.7)
Baseline HBV DNA, IU/ml				
<2,000 (%)*	2,086 (72.4)	108 (68.8)	1,038 (72.9)	940 (72.2)
≥2,000 (%)*	796 (27.6)	49 (31.2)	385 (27.1)	362 (27.8)
Baseline HBeAg				
Negative (%)*	1,421 (66.7)	71 (65.1)	693 (67.3)	657 (66.2)
Positive (%)*	710 (33.3)	38 (34.9)	337 (32.7)	335 (33.8)
Cirrhosis				
No (%)	4,086 (83.5)	202 (69.9)	1,977 (83.4)	1,907 (85.4)
Yes (%)	807 (16.5)	87 (30.1)	395 (16.6)	325 (14.6)
Liver failure				
No (%)	4,749 (97.1)	267 (92.4)	2,296 (96.8)	2,186 (97.9)
Yes (%)	144 (2.9)	22 (7.6)	76 (3.2)	46 (2.1)
NAFLD				
No (%)	4,326 (88.4)	257 (88.9)	2,089 (88.1)	1,980 (88.7)
Yes (%)	567 (11.6)	32 (11.1)	283 (11.9)	252 (11.3)
Alcoholic liver disease				
No (%)	4,715 (96.4)	263 (90.0)	2,273 (95.8)	2,179 (97.6)
Yes (%)	178 (3.6)	26 (9.0)	99 (4.2)	53 (2.4)
Baseline biopsy				
No (%)	4,820 (98.5)	281 (97.2)	2,338 (98.6)	2,201 (98.6)
Yes (%)	73 (1.5)	8 (2.8)	34 (1.4)	31 (1.4)
Hypertension				
No (%)	3,472 (71.0)	181 (62.6)	1,683 (71.0)	1,608 (72.0)
Yes (%)	1,421 (29.0)	108 (37.4)	689 (29.0)	624 (28.0)
Diabetes				
No (%)	4,129 (84.4)	219 (75.8)	1,963 (82.8)	1,947 (87.2)
Yes (%)	764 (15.6)	70 (24.2)	409 (17.2)	285 (12.8)
Baseline NA type (%)				
Adefovir	98 (2.4)	9 (3.8)	50 (2.5)	39 (2.2)
Entecavir	1,257 (31.2)	72 (30.4)	617 (31.1)	568 (31.4)
Lamivudine	126 (3.2)	10 (4.2)	73 (3.7)	43 (2.4)
Telbivudine	4 (0.1)	0 (0)	2 (0.1)	2 (0.1)
Tenofovir	2,045 (50.7)	111 (46.8)	960 (48.4)	974 (53.8)
Combined NAs	500 (12.4)	35 (14.8)	281 (14.2)	184 (10.2)
NAs duration, days (Mean ± SD)	540.2 ± 520.0	724.8 ± 662.1	442.1 ± 461.1	620.6 ± 537.9
Treatment situation at index date (%)				
Off treatment	863 (17.6)	52 (18.0)	389 (16.4)	422 (18.9)
On treatment	4,030 (82.4)	237 (82.0)	1,983 (83.6)	1,810 (81.1)

N, number of patients; SD, standard deviation; HBV, hepatitis B virus; HBeAg, hepatitis e antigen; ALT, alanine aminotransferase; NAFLD, nonalcoholic fatty liver disease; NA, nucleos(t)ide analog.

*Percentages were based on non-missing totals of patients with HBV DNA and HBeAg test results.

### Correlation Between the Peak ALT Level and HCC in the US Cohort

There were 4,560 patients in the US cohort with at least three ALT values after normalization following NA treatment, and 148 patients were diagnosed HCC during the follow-up. A positive, dose-dependent correlation between the peak serum ALT level and HCC was observed (*p* < 0.001) (**Table 2**).

### ALT Elevation, Especially ALT Flare Was a Strong Predictor for HCC in the US Cohort

In the entire study cohort of the US (4,893), there were 172 cases of HCC, 42 in ALT flare group, 94 in minor relapse group, and 36 in normal group.

ALT elevation was associated with a significantly elevated risk of HCC (adjusted HR 2.75, 95%CI 1.89–3.98) compared to those maintained normal ALT (**Supplementary Table 1**). For ALT flare, the risk of developing HCC increased by 8.49-fold (95%CI 5.43–13.26) *versus* the normal group. The results were similar after adjustment for age, gender, baseline cirrhosis, diabetes/hypertension and alcoholic liver disease (HR 7.62, 95% CI 4.85–11.98) (**Table 5**). Similar observations were made in men and women, respectively (**Supplementary Table 2**). The Kaplan–Meier curve confirmed a higher probability of HCC in ALT flare group compare to minor relapse and normal groups (**Figure 2B**).

**Table 5:** Cox proportional hazard model of the association between ALT flare and hepatocellular carcinoma in the US cohort.

	N	HCC	Univariable HR (95% CI)	*p*	Multivariable HR (95% CI)	*p*
N	4,893	172				
ALT pattern						
Normal	2,232	36	–	–	–	–
Minor relapse	2,372	94	1.92 (1.30–2.83)	0.001	2.13 (1.44–3.14)	<0.001
ALT flare	289	42	8.49 (5.43–13.26)	<0.001	7.62 (4.85–11.98)	<0.001
Age						
<40	1,126	14	–	–	–	–
40-60	2,634	81	2.39 (1.35–4.21)	0.003	1.86 (1.05–3.32)	0.034
>60	1,133	77	5.65 (3.20–9.99)	<0.001	4.47 (2.48–8.06)	<0.001
Gender						
Male	3,018	140	–	–	–	–
Female	1,875	32	0.36 (0.25–0.53)	<0.001	0.44 (0.30–0.65)	<0.001
Cirrhosis						
No	4,086	95	–	–	–	–
Yes	807	77	4.23 (3.13–5.71)	<0.001	2.83 (2.05–3.91)	<0.001
Diabetes/hypertension						
No	3,239	95	–	–	–	–
Yes	1,654	77	1.62 (1.20–2.19)	0.002	0.92 (0.67–1.26)	0.596
Alcoholic liver disease						
No	4,715	156	–	–	–	–
Yes	178	16	2.86 (1.71–4.78)	<0.001	1.10 (0.63–1.90)	0.746
Baseline biopsy						
No	4,820	167	–	–		
Yes	73	5	2.00 (0.82–4.88)	0.126		
NA duration, days						
≤180	1,346	60	–	–		
181-365	1,072	37	0.81 (0.54–1.23)	0.322		
>365	2,475	75	0.81 (0.57–1.14)	0.219		
Treatment situation at index day						
Off treatment	863	24	–	–		
On treatment	4,030	148	1.19 (0.77–1.84)	0.428		
NAFLD						
No	4,326	156	–	–		
Yes	567	16	0.85 (0.51–1.42)	0.531		
Liver failure						
No	4,749	166	–	–	–	
Yes	144	6	1.30 (0.57–2.93)	0.532		

ALT, alanine aminotransferase; HCC, hepatocellular carcinoma; HR, hazard ratio; CI, confidence interval; N, number of patients in the group; NAFLD, nonalcoholic fatty liver disease; NA, nucleos(t)ide analog.

*ALT pattern, age, gender, baseline cirrhosis, liver failure, NAFLD, alcoholic liver disease, hypertension/diabetes, biopsy, NA treatment duration and treatment situation on index date were analyzed in the model. Only those factors whose p value <0.1 remained in the final multivariable model.

### Sensitivity Analysis: Associations Between ALT Flare and HCC Among Patients With Drug Resistance Tested

There were 449 patients of China cohort with drug resistance tested. Among these patients, 267 (59.5%) patients were with drug resistance. The top three most frequently used antivirals at baseline in the drug-resistant patients were lamivudine (22.1%), adefovir (14.6%), and telbivudine (13.9%). And majority (92.1%) received rescue therapy. Specifically, 91.1% in the ALT flare group, 93.4% in the minor relapse group, 91.9% in the normal group. The association between ALT flare and HCC remained the same (HR = 2.09) after adjusting for all potential factors and mutation, although it was not statistically significant due to insufficient sample size (**Supplementary Table 3**).

## Discussion

China has one of the highest HBV infection rates among adults in the world, and up to 50% of annual global deaths due to HBV-related complications occur in China (21, 22). To our knowledge, this study is the first to prove ALT flare after normalization predicting HCC occurrence in NA-treated CHB patients. We found that ALT level is significantly associated with HCC, and ALT flare is a strong predictor for HCC occurrence. The risk of HCC was directly proportional to the peak level of ALT. The finding was validated by the analyses in the US Optum Electronic Health Records database.

In our study, the 5-year cumulative incidence rates of ALT flare were 5.6–5.9% in China and the US cohorts, respectively, which is similar to a published 4-year cumulative incidence of 5.7% in CHB patients without NA treatment in the US and Canada (6), although the mechanism of developing ALT flares may be different among treated and untreated CHB patients.

Our study observed that ALT patterns were significantly associated with HCC in CHB patients who had been well controlled by NA. Our findings are in line with published findings that elevated ALT (7, 23), age (24), gender (25), the presence of cirrhosis (24, 26), alcoholic liver disease (25), and comorbidity of diabetes/hypertension (25, 27) are significantly associated with HCC in patients with CHB. The risk of HCC in patients with ALT elevation observed in our study (1.82–2.75-fold) is kind of lower than the 5.8–6.8-fold increases reported in large CHB cohort studies from Taiwan and Hong Kong (7, 8). The reason may be that the population with ALT elevation in those studies referred to or included CHB patients with persistently abnormal ALT. HCC was diagnosed within a short time period (mean around 3 months in China) in patients with ALT elevation, especially ALT flare. This suggests that ALT flare is likely a predictor, rather than a cause, of HCC development.

Parallel analyses conducted in China and US cohorts showed remarkably similar results despite differences in the demographic and clinical features of the two cohorts. Patients with CHB in China were younger, had higher rates of HBeAg positivity and HBV DNA levels ≥2,000, than patients with CHB in the US. These differences probably reflect the different HBV epidemiology in China and the US experienced by adults too old to have been included in infant HBV vaccination programs in either country. Prior to vaccination, the primary route of transmission was perinatal in China, versus horizontal transmission from behaviors relating to lifestyle in the US, as evidenced by the higher rates of HCV and HIV infection among US patients with CHB.

To our knowledge, this study is the first to use real-world data to examine the predictive value of ALT flare for the occurrence of HCC in CHB patients treated with NA in China and the US. Strengths of our study include a large sample size, long term longitudinal follow-up of CHB patients in a natural practice setting, and utilization of the US data which is representative of a different population under a different healthcare system to externally validate our results. We believe our conclusions are solid.

There are some limitations of our study. First, as a retrospective observational research based on real-world practice data, residual confounders are common concerns. Second, because of the limited data, other factors likely to affect the risk of HCC were not included in the study, such as family history, toxic drug use history, etc. However, these factors don’t have known effect on ALT among CHB patients. Thus, they are not likely play confounding roles in our study. Finally, whether abnormal ALT was prolonged or transient after elevation was not studied in the ALT elevation groups.

In conclusion, ALT elevation after normalization, especially ALT flare, is a strong predictor for the development of HCC in CHB patients treated with NA. The results of our large-scale, real-world study conducted in two continents can be used to estimate the risk of incident HCC according to ALT pattern. There was a strong association between ALT flare in NA-treated patients and the development of HCC. Close monitoring of these patients for early detection of HCC is warranted.

## Data Availability Statement

The original contributions presented in the study are included in the article/**Supplementary Material**. Further inquiries can be directed to the corresponding authors.

## Ethics Statement

The studies involving human participants were reviewed and approved by Human Ethics Committee, Ruijin Hospital, Shanghai Jiao Tong University School of Medicine. Written informed consent for participation was not required for this study in accordance with the national legislation and the institutional requirements.

## Author Contributions

HQ, WC, MS, and YZ were involved in the conceptualization. XY and HW were involved in the data curation. MS and YZ provided methodology. YD, BD, XY, HW, MS, and YZ performed the formal analysis. YD and BD wrote the original draft. YS, JT, and PV performed validation. HQ and WC wrote review and editing. All authors contributed to the article and approved the submitted version.

## Funding

This study was funded by the National Natural Science Foundation of Shanghai (20ZR1433500), the Major Project of National Thirteenth Five-year Plan (2017ZX09304016), the National Natural Science Foundation of China (81470867), and the Shanghai Municipal Key Clinical Specialty (shslczdzk1103).

## Conflict of Interest

XF, YZ, MS, HC, and HQ are employees of Janssen Research & Development. YZ and HQ report stock ownership in Johnson and Johnson.

The remaining authors declare that the research was conducted in the absence of any commercial or financial relationships that could be construed as a potential conflict of interest.

## References

[1] Ott JJ Stevens GA Groeger J Wiersma ST Global epidemiology of hepatitis B virus infection: new estimates of age-specific HBsAg seroprevalence and endemicity. Vaccine (2012) 30(12):2212–9. 10.1016/j.vaccine.2011.12.116 22273662

[2] Lok ASF Chronic hepatitis B. N Engl J Med (2002) 346(22):1682–3. 10.1056/NEJM200205303462202 12037146

[3] Xie K Xu C Zhang M Wang M Min L Qian C Yes-associated protein regulates podocyte cell cycle re-entry and dedifferentiation in adriamycin-induced nephropathy. Cell Death Dis (2019) 10(12):915. 10.1038/s41419-019-2139-3 31801948 PMC6892849

[4] Chen W Zheng R Baade PD Zhang S Zeng H Bray F Cancer statistics in China, 2015. CA Cancer J Clin (2016) 66(2):115–32. 10.3322/caac.21338 26808342

[5] Jeng WJ Chen YC Liaw YF Great and rapid HBsAg decline in patients with on-treatment hepatitis flare in early phase of potent antiviral therapy. J Viral Hepatol (2018) 25(4):421–8. 10.1111/jvh.12833 29193573

[6] Brahmania M Lombardero M Hansen BE Terrault NA Lok AS Perrillo RP Association between severe serum alanine aminotransferase flares and hepatitis B e antigen seroconversion and HBV DNA decrease in untreated patients with chronic HBV infection. Clin Gastroenterol Hepatol (2019) 17(12):2541–51. 10.1016/j.cgh.2019.02.005 PMC6905460 30743006

[7] Fung J Cheung KS Wong DK Mak LY To WP Seto WK Long-term outcomes and predictive scores for hepatocellular carcinoma and hepatitis B surface antigen seroclearance after hepatitis B e-antigen seroclearance. Hepatology (2018) 68(2):462–72. 10.1002/hep.29874 29534307

[8] Chen CF Lee WC Yang HI Chang HC Jen CL Iloeje UH Changes in serum levels of HBV DNA and alanine aminotransferase determine risk for hepatocellular carcinoma. Gastroenterology (2011) 141(4):1240–8. 10.1053/j.gastro.2011.06.036 21703214

[9] Lin Y-J Lee M-H Yang H-I Jen C-L You S-L Wang L-Y Predictability of liver-related seromarkers for the risk of hepatocellular carcinoma in chronic hepatitis B patients. PloS One (2013) 8(4):e61448. 10.1371/journal.pone.0061448 23613855 PMC3629190

[10] Hann H-W Wan S Myers RE Hann RS Xing J Chen B Comprehensive analysis of common serum liver enzymes as prospective predictors of hepatocellular carcinoma in HBV patients. PloS One (2012) 7(10):e47687. 10.1371/journal.pone.0047687 23112834 PMC3480412

[11] Pacifico L Ferraro F Bonci E Anania C Romaggioli S Chiesa C Upper limit of normal for alanine aminotransferase: quo vadis? Clin Chim Acta (2013) 422:29–39. 10.1016/j.cca.2013.03.030 23566931

[12] Sarin SK Kumar M Lau GK Abbas Z Chan HL Chen CJ Asian-Pacific clinical practice guidelines on the management of hepatitis B: a 2015 update. Hepatol Int (2016) 10(1):1–98. 10.1007/s12072-015-9675-4 PMC4722087 26563120

[13] European Association for the Study of the Liver EASL 2017 Clinical Practice Guidelines on the management of hepatitis B virus infection. J Hepatol (2017) 67(2):370–98. 10.1016/j.jhep.2017.03.021 28427875

[14] Terrault NA Lok ASF McMahon BJ Chang KM Hwang JP Jonas MM Update on prevention, diagnosis, and treatment of chronic hepatitis B: AASLD 2018 hepatitis B guidance. Hepatology (2018) 67(4):1560–99. 10.1002/hep.29800 PMC5975958 29405329

[15] Lee JK Shim JH Lee HC Lee SH Kim KM Lim Y-S Estimation of the healthy upper limits for serum alanine aminotransferase in Asian populations with normal liver histology. Hepatol (Baltimore Md) (2010) 51(5):1577–83. 10.1002/hep.23505 20162730

[16] Park JH Choi J Jun DW Han SW Yeo YH Nguyen MH Low alanine aminotransferase cut-off for predicting liver outcomes; a nationwide population-based longitudinal cohort study. J Clin Med (2019) 8:1445. 10.3390/jcm8091445 PMC6780691 31514449

[17] The Office for Civil Rights Malin B Guidance regarding methods for de-identification of protected health information in accordance with the Health Insurance Portability and Accountability Act (HIPAA) Privacy Rule. Health Information Privacy (2012) 1–32. Available at: https://www.hhs.gov/hipaa/for-professionals/privacy/special-topics/de-identification/index.html [Accessed Dec 30, 2020].

[18] Rothman KJ GS Modern Epidemiology. 2nd Edition Philadelphia: Lippincott Williams & Wilkins (1998).

[19] Keeffe EB Dieterich DT Han S-HB Jacobson IM Martin P Schiff ER A treatment algorithm for the management of chronic hepatitis B virus infection in the United States: 2008 update. Clin Gastroenterol Hepatol Off Clin Pract J Am Gastroenterol Assoc (2008) 6(12):1315–41. 10.1016/j.cgh.2008.08.021 18845489

[20] Kobashi H Miyake Y Ikeda F Yasunaka T Nishino K Moriya A Long-term outcome and hepatocellular carcinoma development in chronic hepatitis B or cirrhosis patients after nucleoside analog treatment with entecavir or lamivudine. Hepatol Res Off J Japan Soc Hepatol (2011) 41(5):405–16. 10.1111/j.1872-034X.2011.00785.x 21435126

[21] Qiu Y Ren J Yao J Healthy adult vaccination: an urgent need to prevent hepatitis B in China. Hum Vaccin Immunother (2016) 12(3):773–8. 10.1080/21645515.2015.1086519 PMC4964707 26337328

[22] Zhang S Wang F Zhang Z Current advances in the elimination of hepatitis B in China by 2030. Front Med (2017) 11(4):490–501. 10.1007/s11684-017-0598-4 29170919

[23] Wong GL Chan HL Tse YK Yip TC Lam KL Lui GC Normal on-treatment ALT during antiviral treatment is associated with a lower risk of hepatic events in patients with chronic hepatitis B. J Hepatol (2018) 69(4):793–802. 10.1016/j.jhep.2018.05.009 29758335

[24] Papatheodoridis GV Sypsa V Dalekos GN Yurdaydin C Van Boemmel F Buti M Hepatocellular carcinoma prediction beyond year 5 of oral therapy in a large cohort of Caucasian patients with chronic hepatitis B. J Hepatol (2020) 72(6):1088–96. 10.1016/j.jhep.2020.01.007 31981727

[25] Shyu Y-C Huang T-S Chien C-H Yeh C-T Lin C-L Chien R-N Diabetes poses a higher risk of hepatocellular carcinoma and mortality in patients with chronic hepatitis B: a population-based cohort study. J Viral Hepatol (2019) 26(6):718–26. 10.1111/jvh.13077 30739359

[26] Hsu Y-C Yip TC-F Ho HJ Wong VW-S Huang Y-T El-Serag HB Development of a scoring system to predict hepatocellular carcinoma in Asians on antivirals for chronic hepatitis B. J Hepatol (2018) 69(2):278–85. 10.1016/j.jhep.2018.02.032 29551708

[27] Kim NH Cho YK Kim BI Kim HJ Effect of Metabolic Syndrome on the Clinical Outcomes of Chronic Hepatitis B Patients with Nucleos(t)ide Analogues Treatment. Dig Dis Sci (2018) 63(10):2792–9. 10.1007/s10620-018-5165-6 29948568

