# Supplementary material for: ALT Flare Predicts Hepatocellular Carcinoma Among Antiviral Treated Patients With Chronic Hepatitis B: A Cross-Country Cohort Study
Source: Front Oncol. 2021 Jan 21;10:615203. doi: 10.3389/fonc.2020.615203 (PMC7859526; doi:10.3389/fonc.2020.615203)
Supplement: Supplementary file 1 [file DataSheet_1.doc]

Supplementary Material

1. **Supplementary Data**

***Data sources***

***China***

Clinical records of all patients who received healthcare for CHB from January 01, 2010 to December 31, 2018 at Ruijin Hospital, Shanghai Jiao Tong University School of Medicine, China, were de-identified and extracted from the hospital medical records information system. Ruijin Hospital is a tertiary hospital that provides high quality care for and conducts research into multiple diseases areas, including infectious disease. As for most major tertiary hospitals in China, Ruijing Hospital provides integrated services and captures information about outpatient and inpatient episodes, pharmacy, laboratory and diagnostic procedures electronically within the same organization. Diagnoses are coded using the International Classification of Diseases 10th (ICD10) revision and as free text. Healthcare data from Ruijing Hospital is representative of top healthcare in first tier cities in China.

***US***

The Optum PanTher EHR is a healthcare database combining adjudicated claims data with EHR data covering a population of 80 million patients with at least 7 million patients from each US Census region. The database contains a network of more than 140,000 providers at more than 700 hospitals and 7,000 clinics. Optum PanTher EHR is a multi-dimensional database containing de-identified information on outpatient visits, diagnostic procedures, medications, laboratory results, hospitalizations, clinical notes and patient outcomes primarily from Integrated Delivery Networks. The analysis data from January 01, 2010 to December 31, 2018 were extracted from the Optum EHR database.

***Entire study cohorts***

The cohort entry date was the earliest date of an ALT achieving the normal criteria (1^st^ ALT normalization date) with NA treatment. The entire study cohort baseline period was defined as the 12-month period at or prior to the 1^st^ ALT normal date.

**2. Supplementary Tables**

**Supplementary Table 1. Cox proportional hazard model of the association between ALT elevation and hepatocellular carcinoma, in China cohort and in the US cohort**

|  | **N** | **HCC** | **Univariable HR (95% CI)** | ***p*** | **Multivariable HR (95% CI)** | ***p*** |
| --- | --- | --- | --- | --- | --- | --- |
| **Ruijin Hospital, China** | |  |  |  |  |  |
| Normal | 6,720 | 71 | - | - | - | - |
| ALT elevation | 1,432 | 32 | 2.00 (1.32-3.04) | 0.001 | 1.82 (1.19-2.79) | 0.006 |
| **Optum EHR database, the US** | | |  |  |  |  |
| Normal | 2,232 | 36 | - | - | - | - |
| ALT elevation | 2,661 | 136 | 2.54 (1.76-3.68) | <0.001 | 2.75 (1.89-3.98) | <0.001 |

ALT, alanine aminotransferase; HCC, hepatocellular carcinoma; HR, hazard ratio; CI, confidence interval; N, number of patients in the group.

* Patients with ALT flare or minor relapse were included in ALT elevation group.

** Adjusted for age, gender, baseline cirrhosis and diabetes/hypertension in the Ruijin Hospital cohort; adjusted for age, gender, baseline cirrhosis, alcoholic liver disease and diabetes/hypertension in the US cohort.

**Supplementary Table 2. Cox proportional hazard model of the association between ALT flare and hepatocellular carcinoma stratified by gender, in China cohort and in the US cohort**

|  | **N** | **HCC** | **Univariable HR (95% CI)** | ***p*** | **Multivariable HR (95% CI)** | ***p*** |
| --- | --- | --- | --- | --- | --- | --- |
| **Ruijin Hospital, China** | |  |  |  |  |  |
| Males |  |  |  |  |  |  |
| Normal | 4,421 | 59 | - | - | - | - |
| Minor relapse | 788 | 15 | 1.31 (0.74-2.30) | 0.355 | 1.43 (0.80-2.53) | 0.225 |
| ALT flare | 350 | 12 | 2.40 (1.29-4.46) | 0.006 | 2.25 (1.20-4.23) | 0.012 |
| Females |  |  |  |  |  |  |
| Normal | 2,299 | 12 | - | - | - | - |
| Minor relapse | 186 | 2 | 2.02 (0.45-9.02) | 0.358 | 1.80 (0.39-8.33) | 0.451 |
| ALT flare | 108 | 3 | 5.31 (1.50-18.82) | 0.010 | 5.01 (1.40-17.85) | 0.013 |
| **Optum EHR database, the US** | | |  |  |  |  |
| Males |  |  |  |  |  |  |
| Normal | 1,422 | 29 | - | - | - | -- |
| Minor relapse | 1,407 | 77 | 2.15 (1.40-3.30) | <0.001 | 2.31 (1.50-3.55) | <0.001 |
| ALT flare | 189 | 34 | 8.74 (5.32-14.36) | <0.001 | 7.56 (4.57-12.51) | <0.001 |
| Females |  |  |  |  |  |  |
| Normal | 810 | 7 | - | - | - | - |
| Minor relapse | 965 | 17 | 1.48 (0.61-3.60) | 0.393 | 1.47 (0.60-3.59) | 0.402 |
| ALT flare | 100 | 8 | 7.97 (2.88-22.03) | <0.001 | 9.01 (3.25-25.04) | <0.001 |

ALT, alanine aminotransferase; HCC, hepatocellular carcinoma; HR, hazard ratio; CI, confidence interval; N, number of patients in the group.

* Adjusted for age, gender, baseline cirrhosis and diabetes/hypertension in the Ruijin Hospital cohort; adjusted for age, gender, baseline cirrhosis, alcoholic liver disease and diabetes/hypertension in the US cohort.

**Supplementary Table 3. Cox proportional hazard model of the association between ALT flare and hepatocellular carcinoma among patients with drug resistance tested in China cohort**

|  | **N** | **HCC** | **Univariable HR (95% CI)** | ***p*** | **Multivariable HR (95% CI)** | ***p*** |
| --- | --- | --- | --- | --- | --- | --- |
| N | 449 | 13 |  |  |  |  |
| ALT pattern |  |  |  |  |  |  |
| Normal | 281 | 6 | - | - | - | - |
| Minor relapse | 80 | 4 | 1.52 (0.38-6.10) | 0.552 | 1.63 (0.44-6.04) | 0.465 |
| ALT flare | 88 | 3 | 2.09 (0.59-7.44) | 0.254 | 2.09 (0.49-8.89) | 0.316 |
| Drug resistance mutation | |  |  |  |  |  |
| Negative | 182 | 6 | - | - | - | - |
| Positive | 267 | 7 | 0.76 (0.25-2.25) | 0.616 | 0.94 (0.29-3.07) | 0.913 |
| Age |  |  |  |  |  |  |
| <40 | 186 | 0 | - | - | - | - |
| 40-60 | 167 | 4 | 162392951.13 (0.00-Inf) | 0.998 | 197390481.02 (0.00-Inf) | 0.998 |
| >60 | 96 | 9 | 631909041.73 (0.00-Inf) | 0.997 | 1029556550.78 (0.00-Inf) | 0.998 |
| Gender |  |  |  |  |  |  |
| Male | 321 | 12 | - | - | - | - |
| Female | 128 | 1 | 0.20 (0.03-1.51) | 0.117 | 0.13 (0.02-1.02) | 0.052 |
| Cirrhosis |  |  |  |  |  |  |
| No | 359 | 8 | - | - | - | - |
| Yes | 90 | 5 | 2.53 (0.83-7.72) | 0.104 | 0.95 (0.30-3.01) | 0.929 |
| Diabetes/hypertension | |  |  |  |  |  |
| No | 436 | 12 | - | - | - | - |
| Yes | 13 | 1 | 2.84 (0.37-21.83) | 0.317 | 1.38 (0.16-12.05) | 0.772 |
| NAFLD |  |  |  |  |  |  |
| No | 428 | 12 | - | - |  |  |
| Yes | 21 | 1 | 1.94 (0.25-14.94) | 0.524 |  |  |
| NA duration, days | |  |  |  |  |  |
| ≤180 | 162 | 7 | - | - |  |  |
| 181-365 | 74 | 2 | 0.62 (0.13-2.96) | 0.545 |  |  |
| >365 | 213 | 4 | 0.42 (0.12-1.43) | 0.166 |  |  |
| Alcoholic liver disease | |  |  |  |  |  |
| No | 420 | 12 | - | - |  |  |
| Yes | 29 | 1 | 1.11 (0.14-8.52) | 0.923 |  |  |
| Liver failure |  |  |  |  |  |  |
| No | 413 | 11 | - | - |  |  |
| Yes | 36 | 2 | 2.06 (0.46-9.29) | 0.348 |  |  |
| Treatment situation at index date | | |  |  |  |  |
| Off treatment | 70 | 1 | - | - |  |  |
| On treatment | 379 | 12 | 2.02 (0.26-15.56) | 0.500 |  |  |
| Baseline biopsy |  |  |  |  |  |  |
| No | 447 | 13 | - | - |  |  |
| Yes | 2 | 0 | 0.00 (0.00-Inf) | 0.998 |  |  |

ALT, alanine aminotransferase; HCC, hepatocellular carcinoma; HR, hazard ratio; CI, confidence interval; N, number of patients in the group; NAFLD, nonalcoholic fatty liver disease; NA, nucleos(t)ide analogue.
